# Impact of Petty Tyranny on Employee Turnover Intentions: The Mediating Roles of Toxic Workplace Environment and Emotional Exhaustion in Academia

**DOI:** 10.3390/bs14121218

**Published:** 2024-12-18

**Authors:** Javed Iqbal, Zarqa Farooq Hashmi, Muhammad Zaheer Asghar, Attiq Ur Rehman, Hanna Järvenoja

**Affiliations:** 1 School of English Studies, Zhejiang International Studies University, Hangzhou 310058, China; javed@e.gzhu.edu.cn; 2 School of Education, Huazhong University of Science and Technology, 1037, Luoyu Road, Wuhan 430070, China; zarqafarooqhashmi111@gmail.com; 3 Learning and Educational Technology (LET) Research Laboratory, Faculty of Education and Psychology, University of Oulu, 90570 Oulu, Finland; hanna.jarvenoja@oulu.fi; 4 Department of Education, University of Helsinki, 00014 Helsinki, Finland; 5 Department of Cyber Security, School of Cyber Science and Engineering, Huazhong University of Science and Technology, Wuhan 430070, China; attiq_rehman@hust.edu.cn

**Keywords:** petty tyranny, toxic workplace environment, emotional exhaustion, employee turnover intentions, academic institutions

## Abstract

Based on social exchange theory, social psychology theories, and despotic leadership theory, this study explored the impact of petty tyranny on employee turnover intentions. Specifically, the authors examined the mediating effect of toxic workplace environments through emotional exhaustion on this relationship among academicians. The authors surveyed 421 employees using a five-point Likert scale across six universities in Lahore, Pakistan and employed a time-lag research design. Partial least squares structural equation modeling (PLS-SEM) and artificial neural network (ANN) analyses, including performance comparisons of various algorithms, were used to test the relationships among the variables. The analysis results of the study suggested that petty tyranny does not significantly and directly contribute to employee turnover intentions; however, this relationship is positively and significantly mediated by toxic workplace environments and emotional exhaustion. The results indicated that toxic workplace environments and emotional exhaustion also have a direct effect on employee turnover intentions. A serial full mediation was found between petty tyranny and turnover intentions, mediated through a toxic workplace environment and emotional exhaustion. Similarly, results from the performance comparison of various algorithms reveal trade-offs between precision, recall, and processing time, with ZeroR and Stacking REP Tree emerging as the most effective in terms of overall model accuracy. This study contributes to the literature by examining petty tyranny, workplace environment, and emotional exhaustion, highlighting the need to address tyrannical behavior to improve employee retention in academic organizations. Our study offers valuable practical implications, emphasizing addressing these issues to reduce turnover in academic organizations. Our study also provides recommendations for future research directions.

## 1. Introduction

The issue of petty tyranny in workplace settings has gained increasing attention due to its detrimental effects on employee well-being and organizational outcomes [1]. It refers to the insulting and authoritative behavior exhibited by individuals in positions of power, which negatively impacts subordinates’ morale and overall job satisfaction [2]. The toxic behaviors associated with petty tyranny, such as micro-managing, belittling, and demeaning subordinates, contribute significantly to a hostile workplace environment [3]. In academic institutions, where collaboration is important, petty tyranny can be highly damaging. This research explores tyrannical leadership’s dark dyads, and Machiavellian followers contribute to work withdrawal, lower task performance [1], and increased workplace toxicity, leading to emotional exhaustion and higher employee turnover intentions [4].

Petty tyranny is characterized as undesirable behaviors like abuse of authority, micromanagement, belittlement, and withholding of resources, often rooted in a despotic leadership style within organizations [5]. With its subtle yet pervasive effects, petty tyranny provides a valuable lens for examining broader issues related to leadership and workplace well-being [6]. Understanding these dynamics is both timely and impactful, as it addresses pressing organizational challenges that affect individuals and organizations alike. The consequences of petty tyranny can be far-reaching, fostering toxic workplace environments that lead to emotional exhaustion. These adverse outcomes significantly undermine employee morale, reduce productivity, and hinder retention [7]. Over time, such conditions may raise concerns among employees about their ability to meet job objectives effectively, further exacerbating workplace tensions and dissatisfaction [8]. This study focuses on petty tyranny because of its unique relevance to academia, particularly in developing nations where rigid hierarchical structures often amplify the effects of such behavior. Our study explored petty tyranny in these contexts to provide understanding of leadership challenges and workplace well-being to ultimately offer insights to decrease its negative outcomes, such as employee turnover intentions.

Similarly, employee turnover intention is a significant concern for academic institutions [8]. High turnover rates disrupt institutional processes and operations while incurring substantial costs related to recruiting and training new employees [9,10,11,12]. According to the relevant literature, key factors influencing employee turnover intention include salaries, psychological well-being, the quality of mentoring systems, the presence of an ethical climate, fair decision-making processes, job autonomy, and undesirable leadership behaviors such as petty tyranny [13,14]. These factors profoundly affect employee satisfaction and play a significant role in shaping turnover intentions in academic organizations [8].

The workplace environment is a critical factor influencing organizational performance [15]. A toxic workplace environment can lead to unpleasant and harmful experiences for individuals, ultimately impacting their mental and physical well-being [16]. While the effects of such an environment are evident within the organization, only a few employees formally report these incidents, often due to specific personal or professional motives. This silence and avoidant behavior pose significant challenges for researchers attempting to explore this topic [17]. It is widely recognized that victims of workplace violence experience diminished well-being. According to Maslow’s hierarchy of needs, security is a fundamental concern for individuals in any context, and the presence of uncertainty disrupts the attainment of higher-level needs [8]. This lack of security and well-being consequently increases emotional exhaustion and employees’ turnover intentions [8,18].

Emotional exhaustion negatively influences turnover intentions in academic organizations by diminishing job satisfaction, weakening organizational commitment, and impairing productivity and performance [19]. It fosters increased stress, burnout, and a sense of inadequacy, making employees more likely to leave [8,18]. Linked to toxic workplace environments, such as those characterized by micromanagement and abusive leadership, emotional exhaustion exacerbates perceptions of hostility and reduces employees’ ability to cope with workplace challenges [17]. This depletion of emotional resources not only affects mental and physical health but also erodes collegiality and collaboration, which are vital in academic settings [8]. As a result, emotionally exhausted employees often prioritize their well-being by seeking healthier workplace environments, thereby increasing turnover intentions [20,21].

The literature highlights the negative impacts of petty tyranny, toxic workplace environments, and emotional exhaustion, emphasizing their role in increasing employee turnover intentions. This paper builds on calls for further investigation into petty tyranny [8], particularly its implications for leadership [4], and argues for a deeper understanding of how petty tyranny influences employee turnover intentions. It specifically examines the mediating roles of toxic workplace environments and emotional exhaustion in this relationship.

Despotic leadership theory, social exchange theory, and social psychology theories collectively provide a robust framework for exploring the effects of petty tyranny on employee outcomes [8,22]. Despotic leadership theory highlights the dark side of leadership, such as petty tyranny, while social exchange theory explains how inequitable exchanges in organizational contexts foster workplace toxicity and emotional depletion [23,24]. Social psychology theories, such as behavioral intention prediction theories, contribute by analyzing how human behavior is influenced by personal motives, social factors, and situational dynamics [8,23,24]. Together, these theories offer a comprehensive lens to study how petty tyranny creates a toxic workplace environment and emotional exhaustion, ultimately driving employee turnover intentions, particularly in academic settings and developing nations like Pakistan.

The existing literature primarily presents partial models of how undesirable behaviors impact organizational outcomes [13,14]. Petty tyranny is well-documented for its adverse effects [25], including reduced morale [2], increased absenteeism [26], lower productivity [5], diminished creativity [26], heightened stress [27], and weakened organizational trust and loyalty [28]. In academic settings, where collaboration and collegiality are crucial, petty tyranny poses a significant threat by undermining these foundational principles. However, the specific mechanisms linking petty tyranny to turnover intentions remain underexplored, particularly in academic institutions.

This study addresses these gaps by employing a conceptual framework based on despotic leadership theory, social exchange theory, and social psychology theories. The research examines the direct effects of petty tyranny on employee turnover intentions and the mediating roles of toxic workplace environments and emotional exhaustion. Using a quantitative approach with data from 421 faculty members in Pakistan, the study employs PLS-SEM and ANN analyses to test hypotheses and compare performance across algorithms.

The current study contributes in two key ways. First, it examines the adverse psychological mechanisms through which petty tyranny impacts employee turnover intentions in academic settings, specifically highlighting the mediating roles of toxic workplace environments and emotional exhaustion. This understanding is important for identifying the factors that drive increased employee turnover among academicians. Second, the study addresses the persistent gap in understanding how petty tyranny indirectly leads to employee turnover by fostering toxic workplace environments and emotional exhaustion. This knowledge is vital for developing tailored, proactive policies and interventions to address workplace toxicity and mitigate its effects on emotional well-being and retention in academic organizations. By doing so, the study provides actionable recommendations for improving organizational culture and enhancing employee retention. Furthermore, the findings open up new research avenues for exploring how leadership behaviors influence employee turnover intentions, particularly within educational institutions. This clarification is essential to encourage academic institutions to formulate evidence-based policies that minimize the adverse effects of workplace toxicity and improve employee retention.

**Figure 1:**
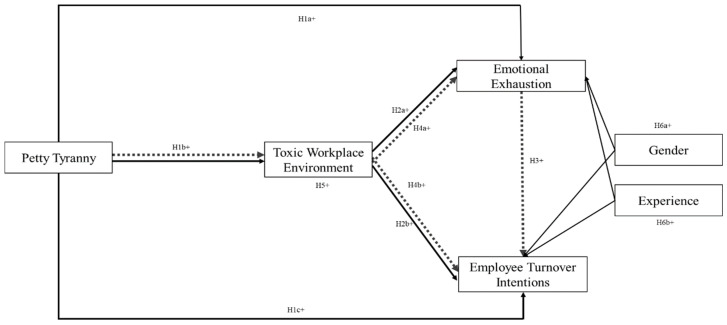
Research Model (solid arrows represent direct relationships, while dashed arrows indicate indirect relationships between variables).

To the best of the authors’ knowledge, this is the first study to explore both the direct and indirect impacts of petty tyranny on employee turnover intentions while also examining the roles of toxic workplace environments and emotional exhaustion as serial mediating variables. This study employed a dual modeling approach, incorporating both structural equation modeling and performance comparison techniques, to measure the relationships between the variables in the research model. Based on these insights, the research model presented in Figure 1 is examined. Therefore, this study seeks to answer the following three research questions (RQs):

**Research Question 1:** How does petty tyranny influence employee turnover intentions among academicians?
**Research Question 2:** How do toxic workplace environments and emotional exhaustion mediate the relationship between petty tyranny and employee turnover intentions?
**Research Question 3:** How does a toxic workplace environment through emotional exhaustion mediate the relationship between petty tyranny and employee turnover intentions?

## 2. Literature Review

### 2.1. Petty Tyranny in Academia: A Background

In academic institutions, particularly in developing nations, petty tyranny has increasingly become a significant issue [29]. Several researchers have examined the impact of petty tyranny on employee turnover intentions, focusing on how such destructive leadership behaviors undermine employee well-being [1,13,14]. Academic institutions rely heavily on collegiality and collaboration for success, making petty tyranny especially harmful to these expectations [30]. For example, leaders who exhibit petty tyrannical behaviors like micromanagement, verbal abuse, and withholding resources can become the cause of creating a toxic workplace environment that fosters disengagement and emotional exhaustion [2]. The concept of petty tyranny, rooted in the broader despotic leadership framework, has gained attention in the recent literature, with scholars exploring its influence on organizational culture, performance, and employee retention across various industries, including higher education [31,32]. Petty tyranny, in contrast to more overt forms of tyrannical leadership, is often subtler and gradually erodes employee morale over time [2]. In academic institutions, this leadership style operates within authoritarian structures, where leaders exert rigid control over subordinates, thereby stifling innovation and creativity [33].

### 2.2. Petty Tyranny

Petty tyranny is referred to as the authoritarian, oppressive, and often abusive behavior exhibited by individuals in positions of power [27]. These individuals engage in actions that undermine the dignity, autonomy, and self-esteem of their subordinates [34]. It typically exerts their control through micromanagement, arbitrary decision-making, public humiliation, and withholding resources or information necessary for task completion [2]. This behavior is rooted in the broader framework of destructive (despotic) leadership, which emphasizes the toxic use of power within organizations and diverges from traditional leadership theories such as transformational or ethical leadership [14]. It is often explained through the lens of social exchange theory, where subordinates’ responses to a toxic workplace environment created by a petty tyrant are seen as a form of withdrawal or disengagement due to the inequitable exchange between supervisors and subordinates [35,36]. The theory highlights that under tyrannical leadership, subordinates feel they give more (effort, loyalty) than they receive (support, recognition), which contributes to a toxic workplace environment. This, in turn, exacerbates emotional exhaustion and increases turnover intentions [13,14]. In this study, petty tyranny is defined as behaviors such as micromanagement, public humiliation, withholding resources, arbitrary decision-making, and verbal abuse by those in power, which create a toxic workplace environment that leads to emotional exhaustion and employee turnover intentions.

### 2.3. Toxic Workplace Environment

Workplace environments can be categorized as collaborative or toxic [15]. A collaborative environment fosters positive connections, while negative interactions between employees and the workplace characterize a toxic environment. A collaborative workplace environment fosters enjoyment, involvement, and positive citizenship behaviors, while a toxic environment is marked by narcissistic behavior, aggressive leadership, antagonistic actions from co-workers and supervisors, as well as ostracism, harassment, and bullying [8]. In toxic workplace environments, psychological and physical imbalances are common, with persistent tension and exhaustion. These conditions often lead to mental and psychological health problems for employees, further exacerbating the adverse effects of workplace toxicity, such as narcissistic behavior, aggressive leadership, and harassment [4,8]. These factors contribute to counterproductive work attitudes and undermine organizational productivity. Building on the literature and the conservation of resources theory, this study highlights crucial elements of a toxic workplace environment, such as harassment, ostracism, and bullying. Ostracism, in particular, refers to the isolation employees feel from their co-workers, supervisors, and stakeholders [4,37]. Harassment refers to the inappropriate handling and threats by supervisors, managers, and peers [8]. Bullying refers to the unfair treatment of employees by individuals or groups in different situations [4]. Workplace ostracism, harassment, and bullying lead to increased employee turnover intentions and reduced job satisfaction [4,8]. Based on the conservation of resources theory and an extensive review of the related literature, these factors contribute to the creation of toxic workplace environments, leading to decreased employee performance, lower work engagement, and reduced job satisfaction [15]. A toxic workplace environment, marked by ostracism, harassment, and bullying, reduces employee performance, work engagement, and job satisfaction. These factors also significantly contribute to emotional exhaustion, amplifying its effects on turnover intentions [4,8]. In the present study, a toxic workplace environment is defined as an organizational setting characterized by harmful behaviors such as ostracism, harassment, and bullying.

### 2.4. Emotional Exhaustion

Emotional exhaustion is defined as a state of chronic physical and emotional depletion resulting from prolonged exposure to stress, particularly in workplace environments [20,21]. It occurs when individuals feel overwhelmed by the demands placed upon them, leading to a sense of fatigue, helplessness, and a reduced ability to cope with work-related challenges [38]. Emotional exhaustion is often the first stage of burnout, where individuals experience a depletion of emotional resources, which eventually impairs their capacity to perform effectively in their professional roles [39]. The concept of emotional exhaustion is based on the conservation of resources theory, which suggests that individuals aim to preserve and safeguard their resources, including energy, time, and emotional well-being, and when these resources are depleted, emotional exhaustion ensues [40]. In workplace settings, emotional exhaustion has been associated with several negative outcomes, such as diminished job satisfaction, decreased organizational commitment, and heightened turnover intentions [20,21]. In this research, emotional exhaustion is conceptualized as a chronic state of mental and physical fatigue caused by prolonged stress, which impairs an individual’s capacity to cope with work demands, eventually leading to employee turnover intentions.

### 2.5. Employee Turnover Intention

Employee turnover intention describes an employee’s inclination to leave their current position and pursue other employment opportunities [8]. This intention often leads to actual turnover behavior, meaning that employees who express the desire to leave are more likely to do so [9,10,11,12]. External factors, such as the availability of alternative employment opportunities, play a crucial role in influencing these intentions. However, internal factors, such as a toxic workplace environment or poor organizational culture, can also strongly influence an employee’s decision to consider leaving [8,41]. In particular, environments characterized by petty tyranny, emotional exhaustion, or lack of support contribute to higher turnover intentions [13,14]. Understanding turnover intention helps organizations anticipate how internal and external factors may contribute to employee turnover, enabling proactive issue resolution. In our study, employee turnover intentions refer to the expressed desire of employees to leave their current organization, driven by dissatisfaction with work conditions and lack of support.

### 2.6. Hypotheses Formulation

The research questions and hypotheses in this study were grounded in social exchange theory, behavioral intention prediction theories, and despotic leadership theory, which collectively provide a robust framework for exploring the impact of petty tyranny on employee turnover intentions [42]. Social exchange theory highlights how inequitable exchanges between leaders and employees foster toxic workplace environments and emotional exhaustion, leading to disengagement and withdrawal behaviors. Behavioral intention prediction theories, such as the theory of planned behavior, explain how employee attitudes, norms, and perceived control shape turnover intentions, emphasizing the mediating roles of emotional exhaustion and workplace toxicity [8,43]. Despotic leadership theory frames petty tyranny as a destructive leadership style that diminishes trust, well-being, and motivation, driving employees to seek alternative employment. These theoretical foundations guided the development of research questions and hypotheses, ensuring alignment with the study’s aim to address gaps in understanding the direct and indirect effects of petty tyranny on employee turnover intentions in academic settings.

The mediators, toxic workplace environment and emotional exhaustion, were chosen based on their theoretical and empirical relevance in explaining the relationship between petty tyranny and employee turnover intentions. Social exchange theory and conservation of resources theory provide the foundation, highlighting how these mediators capture the mechanisms through which leadership behaviors foster workplace toxicity and emotional depletion, ultimately driving employees to disengage and turnover from the organization [8,42].

#### 2.6.1. Petty Tyranny vs. Emotional Exhaustion

According to social exchange theory, employees respond negatively when they perceive inequitable exchanges with their supervisors, often resulting in stress and emotional depletion [44,45]. Emotional exhaustion, a key component of burnout, typically arises when employees experience ongoing stress and mistreatment, such as tyrannical leadership. This behavior creates an environment where employees feel disrespected and undervalued, leading to mental fatigue and reduced coping capacity [27]. Petty tyranny exacerbates this dynamic by fostering feelings of injustice and undermining employee well-being. The research has consistently demonstrated a link between petty tyranny and emotional exhaustion. Another study found that toxic leadership behaviors, including petty tyranny, significantly increase emotional exhaustion among employees due to heightened workplace stress and interpersonal conflict [4]. Despite these findings, limited studies have specifically examined the direct relationship between petty tyranny and emotional exhaustion in academic institutions. Therefore, the current study aims to fill this gap by investigating how petty tyranny influences emotional exhaustion through examining the following hypothesis:

**Hypothesis** **1a** **(H1a):** *Petty tyranny positively influences emotional exhaustion.*

#### 2.6.2. Petty Tyranny vs. Toxic Workplace Environment

Petty tyranny fosters a toxic workplace environment by creating conditions characterized by harassment, bullying, and ostracism, which significantly reduce employee morale and contribute to emotional exhaustion [14]. Despotic leadership behaviors inherent in petty tyranny erode trust and collegiality, leading to a culture of fear and negativity within organizations. This toxic environment not only undermines employee well-being but also diminishes overall organizational productivity. The research supports this connection. For instance, a study [46] demonstrated that toxic leadership behaviors, including petty tyranny, are strongly associated with the development of toxic workplace environments marked by incivility and hostility. Similarly, another study highlighted that tyrannical leadership behaviors significantly increase workplace ostracism and harassment, fostering an atmosphere where employees feel isolated and undervalued [27]. These insights lack evidence from academic settings; therefore, our study explored the connection between petty tyranny and toxic workplace environments through the following hypothesis:

**Hypothesis** **1b** **(H1b):** *Petty tyranny positively influences a toxic workplace environment.*

#### 2.6.3. Petty Tyranny vs. Employee Turnover Intentions

Petty tyranny has been widely recognized as a significant factor contributing to various adverse workplace outcomes, including emotional exhaustion, the development of a toxic workplace environment, and turnover intentions [38]. Multiple studies have demonstrated a positive correlation between petty tyranny and employee turnover intentions, with employees seeking to escape the toxic conditions created by tyrannical leadership [13]. These insights lack evidence from academic settings; therefore, our study explores the connection between petty tyranny and employee turnover intentions through the following hypotheses:

**Hypothesis** **1c** **(H1c):** *Petty tyranny positively influences employee turnover intentions.*

#### 2.6.4. Toxic Workplace Environment vs. Emotional Exhaustion

A toxic workplace environment significantly impacts emotional exhaustion by depleting employees’ psychological resources. The conservation of resources theory explains that when individuals are exposed to ongoing stressors, such as mistreatment, conflict, and lack of support, their emotional reserves are depleted, resulting in exhaustion [47]. The research demonstrated that hostile workplace environments, marked by incivility and harassment, directly contribute to burnout by intensifying employees’ stress levels [4]. Similarly, another study highlighted that employees in toxic environments experience persistent emotional strain, leading to chronic exhaustion and reduced coping abilities [26]. The current study investigates this critical relationship in academic settings through the following hypothesis:

**Hypothesis** **2a** **(H2a):** *Toxic workplace environment positively influences emotional exhaustion.*

#### 2.6.5. Toxic Workplace Environment vs. Employee Turnover Intentions

Toxic workplace environments are key predictors of employee turnover intentions. Persistent negativity, conflict, and hostile behaviors within an organization lead employees to seek alternative employment to escape the psychological toll unsustainable [15]. Studies have consistently linked workplace toxicity to turnover, with demonstrating that environments fostering harassment and ostracism significantly increase employees’ desire to leave [27]. Another study found that toxic work conditions undermine employees’ trust and job satisfaction, making retention strategies ineffective [4,47]. This study explores how toxic workplace environments drive turnover intentions in academic institutions, where subtle forms of toxicity are often overlooked. We formulated the following hypothesis for the study:

**Hypothesis** **2b** **(H2b):** *Toxic workplace environment positively influences employee turnover intentions.*

The relationship between emotional exhaustion and employee turnover intentions is well-documented in organizational research, with emotional exhaustion consistently linked to a weakened attachment to the organization and an increased desire to leave [13,48,49]. Emotional exhaustion, a key component of burnout, depletes employees’ emotional and psychological resources, reducing their ability to handle work-related stress and weakening their commitment to the organization. As emotional exhaustion intensifies, employees are more likely to disengage and view leaving the organization as a viable coping mechanism, making it a key driver of turnover intentions [48]. Previous studies also highlighted emotional exhaustion and turnover intentions, further emphasizing its role in influencing employees’ decisions to leave [50]. Despite this, research has yet to fully explore emotional exhaustion in the context of toxic leadership, particularly in academic settings. This gap emphasizes the need for further investigation into how emotional exhaustion can increase turnover intentions. The following hypothesis was formulated for this study:

**Hypothesis** **3:** *Emotional exhaustion positively influences employee turnover intentions.*

#### 2.6.6. The Mediating Roles of Toxic Workplace Environment

A toxic workplace environment acts as a mediator between petty tyranny and emotional exhaustion by exacerbating stress and resource depletion. Social exchange theory explains that petty tyranny disrupts the balance of employee–employer exchanges, creating a toxic environment characterized by mistreatment and conflict [47]. The research demonstrated that such environments intensify the impact of tyrannical leadership on emotional well-being, making employees more susceptible to burnout [4]. Similarly, a study also highlighted that a toxic environment amplifies emotional strain, reinforcing the link between leadership behaviors and exhaustion [27]. This study explores the mediating role of emotional exhaustion in the relationship between petty tyranny and turnover intentions, especially in academic institutions, where toxicity undermines psychological safety through the following hypothesis:

**Hypothesis** **4a** **(H4a):** *A toxic workplace environment mediates the relationship between petty tyranny and emotional exhaustion.*

A toxic workplace environment mediates the relationship between petty tyranny and employee turnover intentions by increasing employee dissatisfaction and withdrawal behaviors. Destructive leadership creates a climate of hostility, harassment, and exclusion, which heightens turnover intentions [14]. The research revealed that toxic environments act as conduits for the adverse effects of petty tyranny on turnover, as employees perceive leaving as the only viable solution [26]. Furthermore, the research emphasized that workplace toxicity erodes trust and morale, making retention challenging [35,36]. Our study investigates the mediating role of a toxic workplace environment in the relationship between petty tyranny and turnover intentions within academic settings, where such dynamics may lead to severe retention challenges. We formulated the following hypothesis for this purpose:

**Hypothesis** **4b** **(H4b):** *A toxic workplace environment mediates the relationship between petty tyranny and employee turnover intentions.*

#### 2.6.7. The Serial Mediation of Toxic Workplace Environment Through Emotional Exhaustion

Numerous studies have highlighted the detrimental effects of petty tyranny on individual and organizational outcomes. Richard, Boncoeur [14] found that petty tyranny is linked to reduced job satisfaction and increased stress, as subordinates feel disrespected and undervalued. Toxic behaviors such as belittling, unrealistic expectations, and lack of support foster a toxic workplace environment [35,36], which, in turn, leads to emotional exhaustion [46]. Emotional exhaustion is strongly correlated with higher turnover intentions [51]. The relationship between petty tyranny and toxic workplace environments shows that tyrannical leadership fosters fear, mistrust, and low morale, intensifying emotional exhaustion and increasing turnover intentions [52]. However, the serial mediating role of toxic workplace environments through emotional exhaustion in linking petty tyranny to turnover intentions, particularly in academic settings, remains underexplored. This calls for further research into how these factors collectively drive employee turnover intentions, and the following hypothesis was formulated for this study.

**Hypothesis** **5** **(H5):** *A toxic workplace environment through emotional exhaustion serially mediates the relationship between petty tyranny and employee turnover intentions.*

#### 2.6.8. Demographics and Turnover Intention

The previous studies indicate that various physical and psychological traumas do not influence the moderating effect of gender on employment turnover intentions [8]. However, demographic moderators such as gender [53] and experience [54] influence employee turnover intentions. Another study also identified the impact of teacher experience on turnover driven by psychological stress [55]. Consequently, this study also incorporates gender and experience as moderating factors (control variables) for emotional exhaustion and employee turnover intention.

**Hypothesis** **6a** **(H6a):** *The gender demographic has a positive moderating effect on emotional exhaustion and employee turnover intention.*

**Hypothesis** **6b** **(H6b):** *The demographic of work experience has a positive moderating effect on emotional exhaustion and employee turnover intention.*

### 2.7. Research Model

This research model is grounded in three broad theoretical perspectives: social exchange theory, behavioral intention formation theories, and despotic leadership theory. These theories form the basis for examining the relationship between petty tyranny, toxic workplace environments, emotional exhaustion, and employee turnover intentions in academic settings.

Social exchange theory was employed to explain employee turnover intentions in the context of petty tyranny. Theories related to behavioral intention formation, such as the theory of reasoned action and the theory of planned behavior, were also considered, guiding the development of hypothetical relationships between petty tyranny and toxic workplace environments and emotional exhaustion. Despotic leadership theory, particularly concerning tyrannical behavior, illuminated the adverse effects of petty tyranny on employees and how it can lead to increased turnover intentions.

#### 2.7.1. Social Exchange Theory

Social exchange theory was initially developed to explain interpersonal relationships, but in organizational contexts, it has been widely used to understand the employee-employer relationship [42]. According to social exchange theory, relationships thrive on reciprocity, where both parties, in this case, employees and their supervisors or organizations, mutually benefit [8,43]. Employees who perceive favorable exchanges (such as recognition, support, and fair treatment) are more likely to exhibit positive behaviors such as loyalty, organizational commitment, and reduced turnover intentions. On the other hand, when employees feel exploited or underappreciated by a petty tyrant leader, the reciprocity is broken, leading to disengagement, emotional exhaustion, and an increased desire to leave the organization [8].

In this study, petty tyranny breaks the perceived balance in the social exchange. This imbalance fosters a toxic workplace environment, leading to emotional exhaustion, which ultimately drives turnover intentions [8,43]. Social exchange theory thus serves as a foundation for understanding how perceived inequity in the relationship between petty tyrants and their subordinates contributes to emotional strain and withdrawal behavior [8,42].

#### 2.7.2. Behavioral Intention Prediction Theories

Understanding turnover intentions involves understanding human behavior, which is often driven by a combination of personal motives, social factors, and situational influences [8]. The theory of reasoned action and the theory of planned behavior offer key frameworks for understanding how individuals form intentions through their attitudes, subjective norms, and perceived control over their actions [23,24]. The theory of reasoned action posits that employees’ turnover intentions are shaped by their beliefs about the consequences of staying or leaving the organization [56], influenced by their experience with leadership behaviors like petty tyranny [8,43]. The theory of planned behavior expands on this by incorporating the concept of perceived behavioral control [57], recognizing that employees may not always have full control over their decisions to leave [56], particularly in toxic workplace environments where external factors such as lack of job security or fear of retaliation may limit their ability to act on their intentions [8,43]. By applying these intention formation theories, the current study’s authors assumed turnover intention as a product of external factors (e.g., petty tyranny), organizational factors (e.g., toxic workplace environments), and psychological factors (e.g., emotional exhaustion). The theory of planned behavior should be clearly defined as a framework explaining how attitudes, subjective norms, and perceived behavioral control shape intentions and subsequent petty tyranny indirectly influences turnover intentions through emotional and environmental mediators, addressing gaps in the literature, particularly in academic settings [8]. Our study acts on this, emphasizing its relevance in understanding employee turnover intentions.

#### 2.7.3. Despotic Leadership Theory

Despotic leadership must be defined as a leadership style characterized by authoritarian, unethical, and exploitative behaviors, with a justification grounded in its documented impact on workplace well-being and organizational outcomes. Despotic leadership theory, particularly the dark side of leadership, such as petty tyranny, provides a lens through which to explore the negative impact of abusive leadership on employee outcomes [8,22]. Petty tyranny is a leadership style where leaders wield power abusively, creating a hostile and toxic workplace environment that leads to emotional exhaustion and higher turnover intentions [2,27]. The detrimental impact of such leadership on employee morale, engagement, and overall well-being is well-documented, particularly in high-stakes environments like academia, where collaboration and collegiality are very vital [56]. This study proposes that the toxic behaviors of petty tyrants create a toxic workplace environment, which increases emotional exhaustion and turnover intentions. The framework explains petty tyranny’s effects on turnover intentions through the mediating roles of toxic environments and emotional exhaustion, as outlined in Figure 1.

## 3. Methods

### 3.1. Sampling and Participants

To test our hypotheses, we collected data from six universities in Lahore, located in the Punjab province of Pakistan. We first approached the administrative offices to reach out to the deans or directors of major schools within these universities to seek permission for data collection. Once permission was granted, we worked with human resources managers to randomly select approximately 75 teachers from each university. We explained to all participants that their participation would remain anonymous and that their responses would be used solely for research purposes. The surveys were distributed in three waves (from 3 September 2023 to 2 November 2023). At point 1, we sent out questionnaires to 150 employees in university schools. These questionnaires included basic information about the research and assessed the respondents’ perceptions of petty tyranny behaviors, toxic workplace environments, emotional exhaustion, and employee turnover intentions. We received 139 completed questionnaires in the first wave. At time point 2 (four weeks later), we distributed the same survey to individuals who had not responded in the first round, asking them to rate their perceptions of the same constructs. In this round, we received 142 completed responses from faculty members. At time point 3 (four weeks after time point 2), we sent the survey to another group of 150 faculty members, asking them to rate the same constructs. This round yielded 140 completed responses. To ensure data accuracy, we obtained the names of the participants to prevent confusion between different rounds. We also requested that the human resource department provide the names of the employees they evaluated. Once all the questionnaires were collected, we first checked and matched the data. Next, we encoded the data and removed respondents’ names for anonymity.

In total, we collected 421 surveys from employees across 30 schools, with a response rate of 84.2%. On average, there were 14 participants per school. Among the 421 employees, their job positions included teachers, researchers, and administrators. Based on the demographic information in Table 1, 55.1% of the respondents were male (*n* = 232), while 44.9% were female (*n* = 189). Regarding work experience, 26.1% of respondents (*n* = 110) had 1 to 5 years of experience, 37.1% (*n* = 156) had 6 to 10 years of experience, and 36.8% (*n* = 155) had over 11 years of experience. In terms of job roles, 66.5% of the respondents (*n* = 280) were teachers, 21.4% (*n* = 90) were researchers, and 12.1% (*n* = 51) were administrators. This distribution provides a diverse representation of employees across different roles and experiences within the schools surveyed.

### 3.2. Research Design

The research aimed to examine the direct and indirect effects of petty tyranny on employee turnover intentions, with toxic workplace environment and emotional exhaustion as mediators. The study used quantitative methodology and a time-lag survey design to capture perceptions of key constructs over time and minimize common method bias. The research questions and hypotheses were examined using PLS-SEM and ANN analyses to test the hypothesized relationships, ensuring that the methods and analysis provide robust answers to the research questions and offer insights into the mediating mechanisms explored.

This study utilized a stratified random sampling method to ensure the sample was representative of different subgroups within the population. Stratification was based on specific criteria relevant to the research, such as demographic or organizational characteristics, ensuring that each subgroup was adequately represented in the final sample. This method helps to reduce sampling bias and increase the generalizability of the findings. The study utilized a time-lag design, which involved collecting data at different points in time. This approach allowed for a better understanding of changes over time and minimized the risk of common method bias, ensuring more reliable and valid results. Data collection was conducted through a survey using a 7-point Likert scale, where respondents rated their agreement or disagreement with various statements. The Likert scale ranged from 1 (strongly disagree) to 7 (strongly agree), offering a nuanced measurement of attitudes and perceptions. This scale was chosen for its effectiveness in capturing the intensity of respondents’ feelings and attitudes across the variables used in the research model. Advance consent was obtained from all participants before their involvement in the study. Participants were informed about the purpose of the research, the procedures involved, and their rights to confidentiality and withdrawal at any point without penalty. Consent was documented in accordance with ethical guidelines. The study received ethical approval from the Ethics Committee of Zhejiang International Studies University. The research adhered to the ethical standards set forth by the university, which are aligned with the principles outlined in the Declaration of Helsinki.

### 3.3. Instrumentation

In the present study, petty tyranny was the independent variable, and turnover intentions were the dependent variable. Toxic workplace environments and emotional exhaustion were explored as mediators in the relationship between petty tyranny and turnover intentions. A 24-item questionnaire consisting of three parts was developed to measure these variables. The questionnaire adopted reliable and valid constructs and items from prior studies. Five experts reviewed it to recommend contextual adjustments, ensuring content and face validity. Based on their feedback, the items were adapted accordingly. The first section of the questionnaire included details about the study’s purpose, along with statements on anonymity, privacy, and instructions for respondents. The second section gathered demographic information, including gender, work experience, and position. The third part collected data on petty tyranny, toxic workplace environment, social anxiety, and turnover intentions using a 7-point Likert scale (1 = strongly disagree, 7 = strongly agree). Before the final data collection, the reliability and validity of the questionnaire were ensured through a pilot test involving 30 participants with demographic profiles similar to those of the final sample. The pilot study allowed for a practice run of the data analysis, and participants provided feedback, leading to modifications of the questionnaire. The revised version was then used for the final data collection (see Appendix A). 

#### Measures

Petty Tyranny. The items related to petty tyranny were adapted from the work of Kant, Skogstad [58] and Chénard-Poirier, Morin [34]. This section consists of 9 items. Sample statements include “My supervisor often blames me for mistakes, even when they are not my fault” and “My supervisor ignores my concerns or questions about work tasks”. The reliability of the scale was confirmed with a Cronbach’s alpha of 0.875 (Table 2).

Toxic Workplace Environment. The items related to toxic workplace environments were adapted from the study of Rasool, Wang [4] and Iqbal, Asghar [8]. This section consists of 7 items. Sample statements include “My supervisor/co-worker/subordinate never appreciates my contributions or efforts” and “My supervisor/co-worker/subordinate deliberately distances themselves from me or excludes me from team activities”. The reliability of the scale was confirmed with a Cronbach’s alpha of 0.850 (Table 2).

Emotional Exhaustion. The items related to emotional exhaustion were adapted from the work of Klusmann, Richter [59]. This section consists of 6 items. Sample statements include “I feel emotionally drained from my work” and “I feel fatigued when I get up in the morning and have to face another day on the job”. The reliability of the scale was confirmed with a Cronbach’s alpha of 0.817 (Table 2).

Employee Turnover Intentions. The items related to employee turnover intentions were adapted from the study of Iqbal, Asghar [8]. This section consists of 6 items. Sample statements include “I have frequently thought about finding another job” and “I am considering leaving this organization in the near future”. The reliability of the scale was confirmed with a Cronbach’s alpha of 0.780 (see Table 2).

### 3.4. Data Analysis Procedure

The data analysis for this study employed PLS-SEM through SmartPLS 4 and artificial neural networks (ANNs) using MATLAB 7.5 for network design and training and Weka for model evaluation and comparison of machine learning algorithms to examine the relationships between petty tyranny, toxic workplace environment, emotional exhaustion, and employee turnover intentions, including the mediating effects of the toxic workplace environment and emotional exhaustion. The data were initially screened for missing values and outliers, with missing data addressed using mean imputation. Normality tests were performed, and descriptive statistics, including means, standard deviations, skewness, and kurtosis, were calculated to assess data distribution. The measurement model was analyzed using Confirmatory Factor Analysis in PLS-SEM, with a focus on indicator loadings. Internal consistency was evaluated using Cronbach’s Alpha and Composite Reliability. Convergent validity was confirmed through the average variance extracted (AVE), while discriminant validity was verified using the heterotrait–monotrait ratio (HTMT) to ensure the distinctiveness of the constructs.

### 3.5. Common Bias Variance

We employed several remedial techniques to address common method bias in this study. First, the survey utilized a Likert scale with a neutral midpoint to allow respondents to express a neutral or ambivalent stance, reducing the potential for forced extreme responses that can contribute to bias. Employing a time-lag design helped reduce common method bias by collecting data at different points in time, allowing for temporal separation between the measurement of independent and dependent variables. This reduces the risk of respondents’ responses being influenced by consistency motives or the immediate context. Harman’s single-factor test was used as a statistical remedy. Through exploratory factor analysis, the results indicated that no single factor accounted for the majority of the variance, confirming that common method variance was not a significant concern in the data. These combined approaches ensured that the study’s findings were not unduly affected by common method bias.

## 4. Results

### 4.1. Measurement Modeling

We utilized SmartPLS 4 for measurement and structural analyses to assess validity, reliability, and the relationships between the variables in the research model [60]. SmartPLS is known for its statistical efficiency and lower sensitivity to sample size compared to other software commonly used for covariance-based SEM analysis, such as AMOS 26 [61]. This study specifically examined the relationship between petty tyranny and employee turnover intentions, with toxic workplace environment and emotional exhaustion serving as serial mediators. Prior to assessing the direct and indirect relationships, we verified the reliability and validity of the measurement scales used in the research model (see Table 2 and Table 3).

Table 2 provides a detailed assessment of the reliability and validity of the constructs used in this study: petty tyranny, toxic workplace environment, emotional exhaustion, and employee turnover intentions. The reliability of each construct is confirmed through Cronbach’s Alpha (α), with values above the commonly accepted threshold of 0.70, indicating strong internal consistency. Petty tyranny, with an Alpha of 0.875, demonstrates excellent reliability, while the toxic workplace environment (α = 0.850), emotional exhaustion (α = 0.817), and employee turnover intentions (α = 0.780) also exhibit good reliability. The composite reliability (CR) values for each construct further support this, with all CR values exceeding 0.80, confirming that the constructs are reliable and internally consistent. The average variance extracted (AVE) for each construct is above the threshold of 0.50, indicating adequate convergent validity, meaning that the items are well-correlated with their respective constructs.

The variance inflation factor (VIF) values, ranging from 1.387 to 2.471 for all items, are well below the threshold of 5, indicating that there are no multicollinearity issues in the model. This confirms that the items are not excessively correlated, ensuring the robustness of the structural analysis. The item loadings, mostly above 0.6, further validate the measurement model. Overall, the results confirm that the scales used are reliable and valid, with no concerns regarding multicollinearity, strengthening confidence in the structural relationships tested in the study.

Henseler, Ringle [62] criticized the traditional Fornell and Larcker approach for assessing discriminant validity in reflective scales, advocating instead for the heterotrait–monotrait (HTMT) ratio as a more suitable method in PLS-SEM [61,62]. As a result, we used the HTMT approach to assess the discriminant validity of the reflective scales in our research model. According to Hair, Risher [61] and Iqbal, Asghar [8], the HTMT threshold should be below 0.90 to confirm discriminant validity. In our study, all HTMT values fell below this threshold, confirming that the constructs in our model are sufficiently distinct from one another.

The HTMT analysis revealed that the relationships between emotional exhaustion and employee turnover intentions (0.647), and between emotional exhaustion and petty tyranny (0.659), were below the stricter threshold of 0.85, indicating clear discriminant validity. Similarly, the HTMT value between employee turnover intentions and petty tyranny was 0.521, further supporting the distinction between these constructs. The HTMT value between toxic workplace environment and petty tyranny was 0.831, approaching the upper limit but still indicating valid discrimination between the two constructs. The values between toxic workplace environment and emotional exhaustion (0.706) and toxic workplace environment and employee turnover intentions (0.689) were also below the threshold, confirming the discriminant validity across all constructs. We assessed collinearity using the variance inflation factor, where all values ranged from 1.000 to 2.240, well below the threshold of 5, indicating no collinearity concerns in our model.

Similarly, the analysis indicated that the saturated model exhibits a good fit: SRMR (0.075) indicates low residual error. Both d_ULS (1.952) and d_G (0.783) suggest acceptable discrepancies. The Chi-square (698.737) is significant, but given the sample size, it may not be problematic. The NFI (0.686) indicates a reasonable fit, although space for improvement exists.

According to Hair, Risher [61], the r^2^ values range from 0 to 1, indicating the explanatory power of the structural model, with thresholds of 0.75, 0.50, and 0.25 representing substantial, moderate, and weak explanatory power, respectively. Based on the r^2^ values, the toxic workplace environment construct shows an r^2^ value of 0.521, which suggests a moderate explanatory power. This indicates that the model explains 52.1% of the variance in the toxic workplace environment, reflecting a strong predictive ability for this construct. On the other hand, the R^2^ values for emotional exhaustion (0.393) and employee turnover intentions (0.387) indicate that the model explains around 39.3% and 38.7% of the variance in these constructs, respectively. While these values are lower than for toxic workplace environment, they still reflect a moderate level of explanatory power, showing that the model is reasonably effective in predicting emotional exhaustion and turnover intentions based on the latent variables. The adjusted r^2^ values are slightly lower but follow a similar pattern, reinforcing the model’s robustness in explaining the relationships between the constructs. Overall, the r^2^ values suggest that the structural model provides a moderate level of explanatory power for the key constructs under investigation.

The f-square values help assess the effect size of each independent variable on the dependent variables, indicating the proportion of variance explained by each predictor. According to general guidelines, f-square values of 0.02, 0.15, and 0.35 represent small, medium, and large effects, respectively. In this model, petty tyranny has a strong effect on employee turnover intentions, with an f-square value of 1.088, indicating that it significantly contributes to explaining the variance in employee turnover. The f-square value for the influence of petty tyranny on emotional exhaustion is 0.062, reflecting a small effect size, suggesting that petty tyranny contributes to some degree to explaining emotional exhaustion but not as strongly as it does for turnover intentions.

The toxic workplace environment shows a moderate effect on emotional exhaustion (f-square = 0.112), indicating that it plays a substantial role in explaining the variance in emotional exhaustion. However, the toxic workplace environment has a smaller effect on employee turnover intentions, with an f-square value of 0.094, signifying a small to moderate contribution to turnover intentions. Emotional exhaustion has a relatively small effect on turnover intentions (f-square = 0.086), showing that while it influences turnover, its contribution is less significant compared to petty tyranny. In summary, petty tyranny exerts a strong influence on turnover intentions, while the toxic workplace environment has a moderate impact on both emotional exhaustion and turnover, and emotional exhaustion itself has a small but meaningful effect on turnover intentions. This highlights the crucial role of petty tyranny and the toxic workplace environment in shaping employee turnover and emotional responses.

Analysis revealed the descriptive statistics for the constructs used in the study, including petty tyranny, toxic workplace environment, emotional exhaustion, and employee turnover intention. The sample size for all constructs is 421, with each having a minimum value of 1 and a maximum value of 7, based on a Likert scale. The mean values for all constructs are relatively high, indicating that respondents generally experienced high levels of petty tyranny, toxic workplace environments, emotional exhaustion, and turnover intentions. Petty tyranny has a mean of 5.279 with a standard deviation of 0.898, suggesting a moderate level of variability in responses. Similarly, toxic workplace environment (mean = 5.315, standard deviation = 0.901), emotional exhaustion (mean = 5.463, standard deviation = 0.821), and employee turnover intention (mean = 5.472, standard deviation = 1.336) also exhibit a high average level with varying degrees of response variability, especially in turnover intention, which shows the highest variability among the constructs.

Kurtosis values provide insight into the peakedness of the data distribution. Positive kurtosis values indicate that the distributions are more peaked than a normal distribution. Emotional exhaustion has the highest kurtosis (2.891), followed by employee turnover intention (1.982) and toxic workplace environment (1.808), suggesting sharper peaks in these data distributions. Petty tyranny has a relatively lower kurtosis (0.523), indicating that its distribution is closer to normal. Skewness values measure the asymmetry of the distribution. All constructs show negative skewness, indicating a leftward shift in the data, meaning most responses are concentrated toward the higher end of the scale. Emotional exhaustion has the most skewed distribution (−1.738), followed by employee turnover intention (−1.538), toxic workplace environment (−1.423), and petty tyranny (−1.088), which reflects that the majority of participants experienced high levels of these constructs. Overall, these descriptive statistics indicate that participants in the study generally reported high levels of the measured constructs, with varying degrees of peakedness and asymmetry.

The results indicate moderate correlations among all variables. Emotional exhaustion has a moderate correlation with toxic workplace environment (r = 0.582) and employee turnover intentions (r = 0.566), highlighting its mediating role. Similarly, petty tyranny shows a moderate correlation with employee turnover intentions (r = 0.524), suggesting its significant influence on turnover tendencies (see Table 4).

### 4.2. Structural Model Analysis: Direct, Indirect Relationships, Mediation, and Control Variables

**Figure 2:**
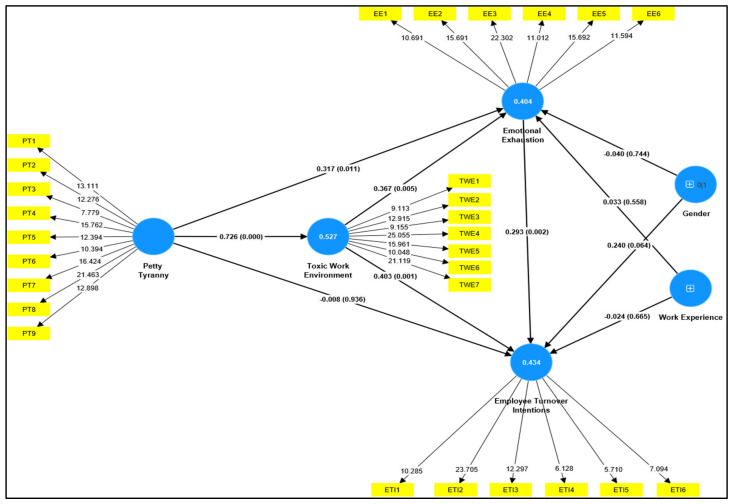
Structural Model.

The results presented in Table 3 highlight the direct relationships between the variables. Petty tyranny has a significant positive impact on emotional exhaustion (β = 0.317, SD = 0.125, t = 2.533, *p* < 0.05), which indicates that petty tyranny directly has a meaningful impact on emotional exhaustion, supporting Hypothesis H1a. Similarly, petty tyranny also significantly influenced the toxic workplace environment (β = 0.726, SD = 0.045, t = 16.123, *p* < 0.05), which reported that petty tyranny has a direct and meaningful impact on the toxic workplace environment, confirming Hypothesis H1b. However, petty tyranny does not have a significant direct effect on employee turnover intentions (β = −0.008, SD = 0.100, t = 0.081, *p* > 0.05), leading to the rejection of Hypothesis H1c. A toxic workplace environment has a significant positive effect on emotional exhaustion (β = 0.367, SD = 0.130, t = 2.822, *p* < 0.05), which indicates that a toxic workplace environment has a direct and meaningful impact on emotional exhaustion, supporting Hypothesis H2a. Similarly, emotional exhaustion has a direct and significant influence on employee turnover intentions (β = 0.293, SD = 0.096, t = 3.045, *p* < 0.05), which indicates that emotional exhaustion has a meaningful impact on employee turnover intentions, supporting Hypothesis H2b. Moreover, a toxic workplace environment also has a significant effect on employee turnover intentions (β = 0.403, SD = 0.117, t = 3.460, *p* < 0.05), which indicates that a toxic workplace environment has a meaningful impact on employee turnover intentions, supporting Hypothesis H3 (see Table 3 and Figure 2).

The indirect relationships in the model show the mediating roles of a toxic workplace environment and emotional exhaustion. Petty tyranny indirectly affects emotional exhaustion through the toxic workplace environment (β = 0.266, SD = 0.099, t = 2.679, *p* < 0.05), supporting Hypothesis H4a. Similarly, the indirect path from petty tyranny to employee turnover intentions through the toxic workplace environment is also significant (β = 0.293, SD = 0.084, t = 3.501, *p* < 0.05), indicating that toxic workplace environment strengthening relationship between petty tyranny and turnover intentions, and supporting Hypothesis H4b. Additionally, the mediating role of both toxic workplace environment and emotional exhaustion in the relationship between petty tyranny and employee turnover intentions is significant (β = 0.078, SD = 0.041, t = 1.893, *p* < 0.05), supporting Hypothesis H5.

The results suggest partial mediation in the model, as petty tyranny’s effect on emotional exhaustion and employee turnover intentions is mediated by the toxic workplace environment and emotional exhaustion. Specifically, the significant indirect effect of petty tyranny on emotional exhaustion and employee turnover intentions through the toxic workplace environment (H4a, H4b) indicates that the toxic environment explains part of the relationship between petty tyranny and employee turnover intention. The final indirect path, where both the toxic workplace environment and emotional exhaustion mediate the relationship between petty tyranny and employee turnover intentions (H5), suggests a more complex mediation process, implying that petty tyranny first creates a toxic workplace environment, leading to emotional exhaustion, which ultimately increases turnover intentions (see Table 3 and Table 4; Figure 2).

In the second part of Table 3, the influence of control variables such as gender and work experience on emotional exhaustion and employee turnover intentions is examined. The results indicate that neither gender nor work experience has a significant effect on emotional exhaustion or employee turnover intentions. Specifically, gender does not significantly influence emotional exhaustion (β = −0.040, SD = 0.122, t = 0.327, *p* > 0.05) or employee turnover intentions (β = 0.240, SD = 0.130, t = 1.852, *p* > 0.05). Similarly, work experience does not significantly affect emotional exhaustion (β = 0.033, SD = 0.056, t = 0.586, *p* > 0.05) or employee turnover intentions (β = −0.024, SD = 0.056, t = 0.433, *p* > 0.05). Thus, no control variable shows a direct or significant influence on either emotional exhaustion or turnover intentions. Thus, H6a–H6d was not supported (see Table 3 and Figure 2).

### 4.3. Machine Learning

**Figure 3:**
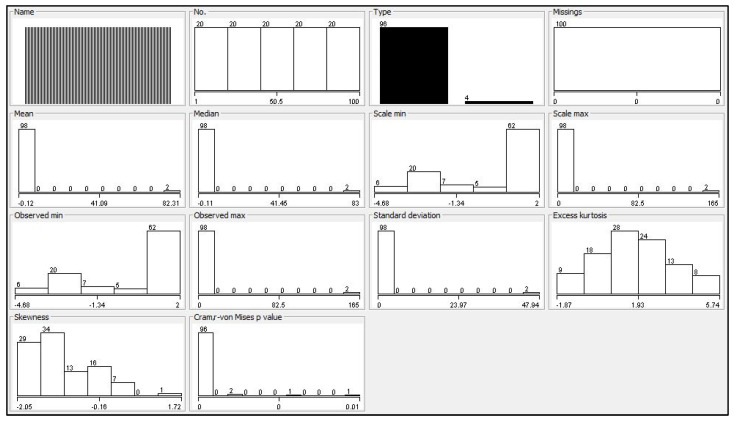
Statistical Summary and Data Distribution Overview.

Figure 3 provides a comprehensive statistical overview of the dataset, using multiple histograms and plots to illustrate key characteristics such as data distribution, central tendencies (mean, median), variability (standard deviation), and shape (skewness, kurtosis). It highlights a uniform distribution across categories like frequency of observations and data type, with no missing values. The dataset shows a normal distribution in terms of mean and median, with some extreme values in observed minimum and maximum. Skewness and kurtosis plots indicate slight asymmetry and varying degrees of tail heaviness, while the Cramér-von Mises *p*-value suggests a minimal deviation from the expected distribution. Overall, the figure provides crucial insights for data exploration and analysis.

**Figure 4:**
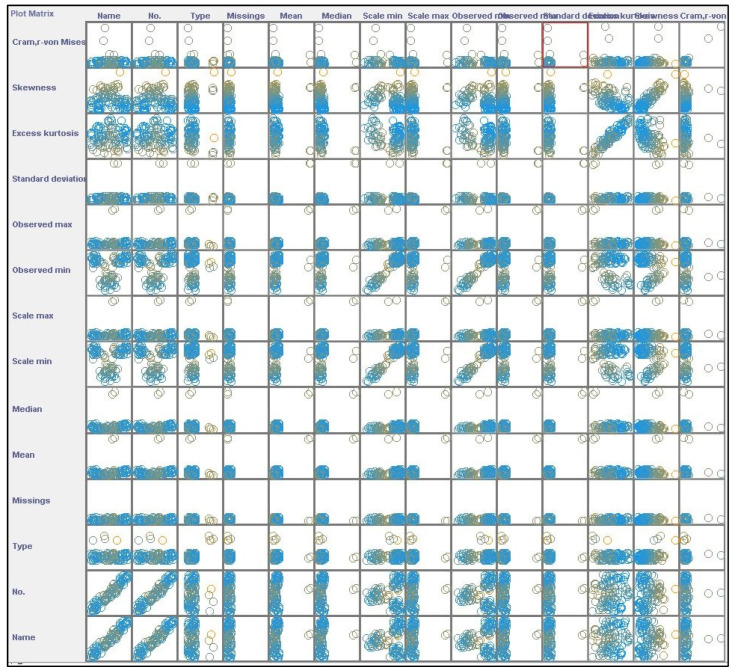
Pairwise Relationship Plot Matrix of Dataset Variables.

Figure 4 presents a scatter plot matrix, showing pairwise relationships between various dataset variables such as name, type, mean, median, skewness, and more. Each off-diagonal cell contains a scatter plot depicting interactions between two variables, while diagonal cells display identity plots or distributions of individual variables. Circular markers in the scatter plots, varying in color, represent individual data points and highlight potential patterns, correlations, or outliers. The matrix helps identify relationships, with dense clusters suggesting correlations and scattered points indicating weaker interactions. Key insights include visible clusters between summary statistics like mean and median, as well as potential relationships between skewness, standard deviation, and kurtosis. Overall, the matrix serves as a tool for exploratory data analysis, helping to visually assess correlations before more complex analyses are performed.

### 4.4. Artificial Neural Network (ANN)

**Figure 5:**
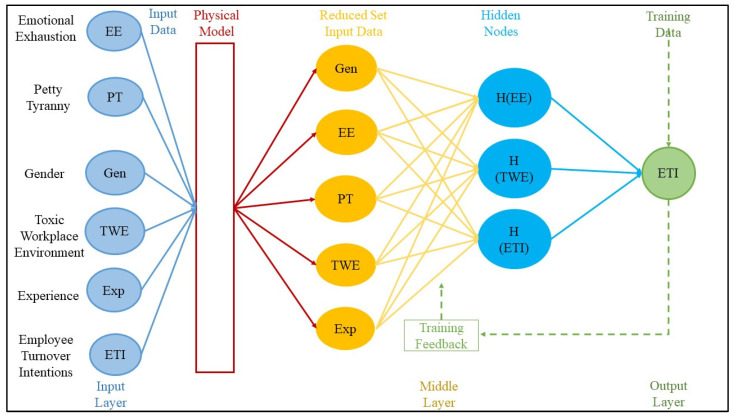
Artificial Neural Network Model for Predicting Employee Turnover Intentions.

Figure 5 illustrates a multi-layer ANN designed to predict employee turnover intentions based on various workplace and employee factors. The network includes an input layer consisting of six variables: emotional exhaustion, petty tyranny, gender, toxic workplace environment, experience, and employee turnover intentions. These inputs pass through a physical model that reduces them to four critical factors, which are then processed by hidden nodes that interpret and integrate the data. A feedback loop suggests supervised learning, allowing the model to adjust based on training data. The final output, generated by the output layer, is a prediction of employee turnover intentions. This ANN model enables the analysis of complex relationships between variables, such as emotional exhaustion, workplace toxicity, and leadership behavior, to predict turnover intentions within an organization.

### 4.5. Performance Comparison of Machine Learning Algorithms

Table 5 compares the performance of four machine learning algorithms—ZeroR decision table, stacking REP tree, IBK, and input mapped classifier—based on key metrics. ZeroR decision table achieved the highest precision (0.980), recall (0.988), and accuracy (93%), making it the most effective at classification despite incorrectly classifying 25 instances. Stacking REP tree had a good balance with 91% accuracy and the fastest processing time (0.8 ms), though its recall (0.872) was slightly lower. IBK showed the lowest performance in terms of correctly classified instances (4), recall (0.864), and accuracy (89%) but still maintained a decent F1-Score (0.885). The input mapped classifier also performed well with 90% accuracy and balanced precision (0.920) and recall (0.859), though it did not outperform the others in any specific metric. Similarly, results from the performance comparison of various algorithms reveal trade-offs between precision, recall, and processing time, with ZeroR and Stacking REP Tree emerging as the most effective in terms of overall model accuracy.

## 5. Discussion

This study examined the effects of petty tyranny on employee turnover intentions within Pakistani academic institutions, considering the mediating roles of a toxic workplace environment and emotional exhaustion. A revised and synthesized research model was developed based on the findings. While similar research has been conducted in developed and emerging countries, few studies have explored this issue in developing nations [14,27]. There is a pressing need for such research in higher education, as addressing petty tyranny can help reduce employee turnover intentions in Pakistani academic institutions. To the best of our knowledge, this research is one of the first to investigate the influence of petty tyranny on turnover intentions in Pakistan’s academic sector, particularly using toxic workplace environments and cognitive distraction as mediators. The study’s findings are discussed in Section 4.

We measured the direct relationship between petty tyranny and emotional exhaustion, toxic workplace environment, and employee turnover intention. The results indicated that petty tyranny significantly contributed to emotional exhaustion and a toxic workplace environment, thereby supporting Hypotheses H1a and H1b. These findings align with the previous research by Guo, Khassawneh [27] and Richard, Boncoeur [14], which demonstrated that petty tyranny increases emotional exhaustion and workplace toxicity by fostering stress and negative interpersonal dynamics. Similarly, studies by Rasool, Wang [4] and Boudrias, Rousseau [26] have highlighted that tyrannical leadership fosters hostile environments, exacerbating emotional fatigue and disengagement among employees. However, the results did not show a direct relationship between petty tyranny and employee turnover intention, meaning that H1c was not supported. This suggests that while petty tyranny creates a toxic workplace environment and contributes to emotional exhaustion, these factors may indirectly lead to employee turnover intentions rather than having a direct impact. This finding echoes the conclusions of Iqbal, Asghar [8], who highlighted that the indirect effects of tyrannical (despotic) leadership through mediating variables like toxic environments play a more significant role in influencing turnover intentions.

We also explored the direct connection between a toxic workplace environment, emotional exhaustion, and turnover intentions. The outcomes of our study indicated that a toxic workplace environment has a direct connection with both emotional exhaustion and turnover intentions, thereby supporting Hypotheses H2a and H2b. These findings are consistent with the research of Rasool, Wang [4], who found that toxic workplace environments lead to higher levels of emotional exhaustion due to increased stress and interpersonal conflict. Similarly, Boudrias, Rousseau [26] reported that hostile and unsupportive workplace conditions significantly contribute to burnout and emotional fatigue, which in turn heightens employees’ intentions to leave. Gravili, Manuti [63] also highlighted that toxic leadership and workplace negativity create an environment where employees experience emotional depletion, increasing their likelihood of seeking alternative employment. Furthermore, the work of Wang, Zaman [46] supported these findings by demonstrating that environments characterized by incivility and bullying not only cause emotional exhaustion but also directly drive employees’ turnover intentions.

Our study explored the direct connection between emotional exhaustion and employee turnover intentions. The results indicated that emotional exhaustion significantly increases employee turnover intentions, thereby supporting Hypothesis H3. These findings are consistent with previous studies that have reported similar outcomes. For instance, Akhtar and Shaukat [13] found that emotional exhaustion, as a core element of burnout, leads to a diminished sense of attachment to the organization, driving employees to consider leaving. Similarly, the study by Lee, Richards [51] demonstrated that emotional exhaustion reduces employees’ psychological resilience and ability to cope with work demands, which increases their intentions to seek alternative employment. Furthermore, Parray, Islam [38] confirmed that emotional exhaustion exacerbates employees’ desire to leave their jobs, particularly when they feel overwhelmed by continuous stress. Research by Feng and Cui [48] showed that emotional exhaustion plays a mediating role between workplace stressors and turnover intentions.

We also measured the mediating role of the toxic workplace environment in the relationship between petty tyranny, emotional exhaustion, and employee turnover intentions. The results indicated that the toxic workplace environment mediates the relationship between petty tyranny and both emotional exhaustion and employee turnover intentions, thereby supporting Hypotheses H4a and H4b. These findings are consistent with earlier studies that have highlighted the mediating role of a toxic workplace environment in similar contexts. For example, Guo, Khassawneh [27] demonstrated that petty tyranny fosters a toxic work atmosphere, which subsequently leads to increased emotional exhaustion among employees. Similarly, Richard, Boncoeur [14] found that a toxic workplace environment serves as a crucial mediator, amplifying the negative effects of tyrannical leadership on employees’ emotional well-being and turnover intentions. Rasool, Wang [4] also confirmed that workplace toxicity created by destructive leadership behaviors intensifies stress and emotional exhaustion, which ultimately drives employees to leave the organization. Boudrias, Rousseau [26] highlighted the role of a toxic environment in translating tyrannical leadership into adverse outcomes like burnout and higher turnover intentions, further validating the mediating effect.

We also explored the serial mediating role of the toxic workplace environment, through emotional exhaustion, in the relationship between petty tyranny and employee turnover intentions, which supported Hypothesis H5. The findings indicated that a toxic workplace environment, when coupled with emotional exhaustion, serves as a serial mediator, amplifying the impact of petty tyranny on employee turnover intentions. These results are consistent with previous studies that have identified a similar serial mediation process. For instance, Wang, Zaman [46] found that a toxic workplace environment exacerbates emotional exhaustion, which, in turn, strengthens the link between tyrannical leadership and the desire to leave the organization. Similarly, Richard, Boncoeur [14] demonstrated that workplace toxicity coupled with emotional fatigue creates a cumulative effect, leading to higher turnover intentions among employees subjected to petty tyranny. The study by Rasool, Wang [4] also supported this view, highlighting that toxic work conditions cause emotional exhaustion and serve as a mechanism through which tyrannical behaviors push employees toward leaving. Moreover, Boudrias, Rousseau [26] emphasized the combined role of emotional exhaustion and workplace toxicity in facilitating the adverse effects of destructive leadership on turnover intentions, further confirming the serial mediating role.

Based on the above discussion, we deduced that collaboration is a cornerstone of academic and organizational success, and toxic workplace environments created by petty tyranny severely hinder this dynamic. By addressing such behaviors and prioritizing emotional well-being, institutions can create a culture of trust and teamwork, ultimately enhancing high performance and ensuring sustainable growth.

## 6. Conclusions

The synthesized research framework, grounded in social exchange, social psychology, and despotic leadership theories, was used to test the hypothesized relationships among the key variables. The results confirmed that petty tyranny serves as a significant source of emotional exhaustion and contributes to a toxic workplace environment within academic settings in Pakistan. Furthermore, the findings indicate that a toxic workplace environment exacerbates emotional exhaustion and increases employee turnover intentions. The toxic workplace environment intensifies the relationship between petty tyranny, emotional exhaustion, and turnover intentions. Moreover, emotional exhaustion, induced by the toxic environment, further strengthens the link between petty tyranny and employee turnover intentions.

The conclusions of this study were derived from the findings and discussion. The results hold particular significance as the research was conducted during an unprecedented period in Pakistan’s academic sector. Despite these unique circumstances, the key outcomes can be summarized and interpreted as follows: First, high levels of petty tyrannical behaviors can significantly predict emotional exhaustion and a toxic workplace environment. Second, a toxic workplace environment can contribute to increased emotional exhaustion and higher employee turnover intentions. Third, a toxic workplace environment alone can lead to employee turnover intentions. Fourth, a toxic workplace environment plays an intervening role in the relationship between petty tyranny, emotional exhaustion, and turnover intentions. Finally, the toxic workplace environment has a serial mediating effect through emotional exhaustion, further strengthening the relationship between petty tyranny and employee turnover intentions in the academic sector.

### 6.1. Theoretical Contributions

This study makes several theoretical contributions to the literature on leadership, workplace environments, and employee turnover, particularly within the academic context of a developing nation like Pakistan. Firstly, it extends the understanding of petty tyranny by demonstrating its significant role in fostering toxic workplace environments and emotional exhaustion, highlighting a previously underexplored mechanism in academia. This research introduces toxic workplace environments and emotional exhaustion as critical mediating variables, thereby providing a more nuanced view of how tyrannical leadership indirectly influences employee turnover intentions. By examining these relationships in the Pakistani academic context, where similar studies are sparse, this research contributes to the global discourse on leadership behavior and employee outcomes, offering insights into how contextual factors in developing nations may influence these dynamics. Furthermore, this study validates the serial mediation model, emphasizing the compounded effects of workplace toxicity and emotional exhaustion on turnover intentions, thus enriching the theoretical understanding of the indirect pathways through which destructive leadership can impact employee retention. These findings align with and expand upon existing leadership theories, such as social exchange theory and despotic leadership theory, by contextualizing them within a toxic workplace framework.

### 6.2. Practical Implication

Our study also offers valuable practical implications. Firstly, considering the significant impact of petty tyrannical behaviors and their detrimental effects on employees’ emotional health, academic organizations should implement strategies to discourage toxic leadership practices. Specifically, academic institutions should foster a culture of respect and fairness by promoting leadership development programs that focus on ethical and compassionate leadership behaviors. Training leaders to recognize and address their negative behaviors can prevent the emergence of petty tyranny in the workplace. Secondly, our study highlights the role of the toxic workplace environment as a driver of emotional exhaustion and employee turnover intentions. To address this, organizations should prioritize creating a healthy workpalce environment by actively monitoring employee well-being and promoting open communication. Institutions can introduce stress management workshops and employee assistance programs aimed at reducing emotional exhaustion and enhancing job satisfaction. Moreover, our outcomes also suggested that a toxic workplace environment directly contributes to employee turnover intentions; therefore, academic institutions should invest in wellness programs and foster a positive culture to retain employees. Regular feedback and recognition initiatives can make employees feel valued and reduce turnover. Since a toxic environment mediates the link between petty tyranny, emotional exhaustion, and turnover, organizations should introduce mediation and conflict resolution services to address issues early. Emotional exhaustion, which worsens the impact of petty tyranny on turnover, can be mitigated by providing mental health support, counseling, and promoting work–life balance, ultimately improving employee retention. To minimize petty tyranny, toxic workplace environments, and emotional exhaustion in developing countries like Pakistan, academic institutions should implement leadership training programs, enforce anti-bullying and harassment policies, and establish mental health support systems. Regulatory frameworks must emphasize workplace psychological safety, with mechanisms for accountability and anonymous feedback to identify and address issues early. Additionally, awareness campaigns and culturally sensitive policies can promote inclusive workplace practices, fostering respectful and collaborative environments.

### 6.3. Limitation and Future Research

Despite its valuable contributions, this study has several limitations that provide opportunities for future research. First, the research was conducted within the academic sector in Pakistan, limiting the generalizability of the findings to other sectors and countries. Future studies could examine the relationships between petty tyranny, workplace toxicity, emotional exhaustion, and turnover intentions across different industries and cultural contexts to validate and extend the findings. Second, the study relied on time-lag data, which limits the ability to establish causality between the variables. Longitudinal studies are recommended to better understand the temporal dynamics of how petty tyranny and toxic workplace environments evolve over time and impact employee outcomes. Third, the study focused on academic institutions, leaving unexplored the potential differences between public and private sector organizations. Future research could investigate how organizational type influences the effects of toxic leadership and workplace environments. Lastly, this research primarily examined the mediating roles of toxic workplace environments and emotional exhaustion; however, other mediators or moderators such as organizational support, leadership development programs, and employee resilience could be explored in future studies to provide a more comprehensive understanding of how to mitigate the adverse impacts of petty tyranny. Expanding on these aspects will help develop more effective strategies for improving workplace culture and employee well-being.

## Figures and Tables

**Table 1:** Demographic Information of Respondents.

Variables	Category	Frequency (*f*)	Percentage
Gender	Male	232	55.1%
	Female	189	44.9%
	Total	421	100%
Work Experience	Year 1 to 5	110	26.1%
	Years 6 to 10	156	37.1%
	Year 11 and above	155	36.8%
	Total	421	100%
Positions	Teaching	280	66.5%
	Research	90	21.4%
	Administrators	51	12.1%
	Total	421	100%

**Table 2:** Reliability, Validity, and Multicollinearity Assessment of Constructs.

**Petty Tyranny**	**Items**	**VIF**	**Loading**
Alpha = 0.875 CR rho_a = 0.878 CR rho_c = 0.901 AVE = 0.534	PT1	1.713	0.713
	PT2	1.560	0.664
	PT3	1.387	0.608
	PT4	1.728	0.733
	PT5	1.868	0.737
	PT6	1.682	0.684
	PT7	1.893	0.751
	PT8	2.019	0.772
	PT9	1.886	0.743
**Toxic Workpalce Environment**	**Items**	**VIF**	**Loading**
Alpha = 0.850 CR rho_a = 0.856 CR rho_c = 0.887 AVE = 0.531	TWE1	1.525	0.649
	TWE2	1.610	0.700
	TWE3	1.437	0.670
	TWE4	2.471	0.849
	TWE5	1.895	0.758
	TWE6	1.567	0.641
	TWE7	2.043	0.806
**Emotional Exhaustion**	**Items**	**VIF**	**Loading**
Alpha = 0.817 CR rho_a = 0.825 CR rho_c = 0.868 AVE = 0.524	EE1	1.644	0.662
	EE2	2.465	0.774
	EE3	2.242	0.802
	EE4	1.784	0.657
	EE5	2.113	0.743
	EE6	1.684	0.692
**Employee Turnover Intentions**	**Items**	**VIF**	**Loading**
Alpha = 0.780 CR rho_a = 0.799 CR rho_c = 0.871 AVE = 0.692	ETI1	1.528	0.693
	ETI2	1.657	0.802
	ETI3	1.683	0.756
	ETI4	1.537	0.693
	ETI5	1.681	0.648
	ETI6	1.562	0.628

**Table 3:** Structural Model Results: Direct, Indirect Relationships, and Control Variables.

**Hypotheses**	**Direct Relations**	**Coefficients**	**Mean**	**SD**	**T Statistics**	***p* Values**	**Decision**
H1a:	PT → EE	0.317	0.315	0.125	2.533	0.011	Sig
H1b:	PT → TWE	0.726	0.73	0.045	16.123	0.000	Sig
H1c:	PT → ETE	−0.008	−0.004	0.1	0.081	0.936	InSig
H2a:	TWE → EE	0.367	0.379	0.13	2.822	0.005	Sig
H2b:	EE → ETE	0.293	0.297	0.096	3.045	0.002	Sig
H3:	TWE → ETE	0.403	0.405	0.117	3.46	0.001	Sig
**Hypotheses**	**Indirect Relations**	**Coefficients**	**Mean**	**SD**	**T statistics**	***p* values**	**Decision**
H4a:	PT → TWE → EE	0.266	0.278	0.099	2.679	0.007	Sig
H4b:	PT → TWE → ETI	0.293	0.294	0.084	3.501	0	Sig
H5:	PT → TWE → EE → ETI	0.078	0.082	0.041	1.893	0.04	Sig
**Hypotheses**	**Control Variables**	**Coefficients**	**Mean**	**SD**	**T statistics**	***p* values**	**Decision**
H6a:	Gender → EE	−0.04	−0.04	0.122	0.327	0.744	InSig
H6b:	Gender → ETI	0.24	0.235	0.13	1.852	0.064	InSig
Control variable:	Work Experience → EE	0.033	0.031	0.056	0.586	0.558	InSig
Control variable:	Work Experience → ETI	−0.024	−0.022	0.056	0.433	0.665	InSig

Note: PT; petty tyranny, TWE; toxic workpalce environment, EE; emotional exhaustion, ETI; employee turnover intentions.

**Table 4:** Correlation matrix.

Variables	1	2	3	4
1. Petty Tyranny	-			
2. Employee Turnover Intentions	0.524 **	-		
3. Toxic Workplace Environment	0.463 **	0.471 **	-	
4. Emotional Exhaustion	0.482 **	0.566 **	0.582 **	-

** Correlation is significant at the 0.01 level (2-tailed).

**Table 5:** Performance Comparison of Machine Learning Algorithms Based on Classification Metrics.

Algorithm	Time (ms)	Correctly Classified	Incorrectly Classified	Precision	Recall	F1-Score	Accuracy
ZeroR Decision Table	0.9 ms	5	25	0.980	0.988	0.903	93%
Stacking REP Tree	0.8 ms	7	22	0.950	0.872	0.895	91%
IBK	0.11 m	4	21	0.930	0.864	0.885	89%
Input Mapped Classifier	0.9 ms	4	18	0.920	0.859	0.880	90%

## Data Availability

Data are contained within the article.

## Appendix A. Questionnaire

**Petty Tyranny**
My supervisor often blames me for mistakes, even when they are not my fault.
My supervisor regularly takes credit for my work without acknowledging my contributions.
My supervisor encourages unethical behavior to meet organizational goals.
My supervisor mocks me in front of others under the guise of humor.
My supervisor frequently displays anger and frustration when I or others do not meet goals.
My supervisor ignores my concerns or questions about work tasks.
My supervisor tells me to follow instructions without offering input or feedback.
My supervisor accuses me of not being a team player without justification.
My supervisor encourages unhealthy competition among employees by rewarding those who criticize or undermine others.
**Toxic Workplace Environment**
My supervisor/co-worker/subordinate never appreciates my contributions or efforts.
My supervisor/co-worker/subordinate often speaks rudely to me in front of others.
My supervisor/co-worker/subordinate frequently makes inappropriate jokes, even when I am uncomfortable.
My supervisor/co-worker/subordinate assigns me tasks that are well outside my skillset, without proper support.
My supervisor/co-worker/subordinate often asks me inappropriate personal or intimate questions.
My supervisor/co-worker/subordinate deliberately distances themselves from me or excludes me from team activities.
My supervisor/co-worker/subordinate consistently overlooks or fails to acknowledge my contributions
**Emotional Exhaustion**
I feel emotionally drained from my work.
I feel used up at the end of the workday.
I feel fatigued when I get up in the morning and have to face another day on the job.
I feel strained from the demands of my work, especially when interacting with others.
I feel mentally and emotionally burned out from my work.
I feel emotionally frustrated and overwhelmed by my job.
**Turnover Intentions**
In the past year, I have seriously considered leaving my job.
I have frequently thought about finding another job.
I often think about quitting my job.
I have actively searched for alternative job opportunities.
I am considering leaving this organization in the near future.
In the past year, I thought about quitting my job because of work-related issues.
